# Routine vaccination for influenza and pneumococcal disease and its effect on COVID-19 in a population of Dutch older adults

**DOI:** 10.1016/j.jvacx.2023.100344

**Published:** 2023-07-06

**Authors:** Esther J.M. Taks, Konstantin Föhse, Simone J.C.F.M. Moorlag, Jacobien Hoogerwerf, Reinout van Crevel, Cornelis H. van Werkhoven, Mihai G. Netea, Jaap ten Oever

**Affiliations:** a Department of Internal Medicine, Radboud University Medical Center, Nijmegen, The Netherlands; b Radboud Center for Infectious Diseases, Radboud University Medical Center, Nijmegen, The Netherlands; c Julius Center for Health Sciences and Primary Care, University Medical Center Utrecht, Utrecht, The Netherlands; d Department for Immunology and Metabolism, Life and Medical Sciences Institute (LIMES), University of Bonn, Germany

**Keywords:** Influenza vaccine, Pneumococcal vaccine, Older adults, COVID-19, Trained immunity

## Abstract

**Objectives:**

Protective heterologous beneficial effects of vaccines have been reported, and in this study we aimed to assess the impact of routine pneumococcal and influenza vaccination on the incidence and symptom duration of COVID-19 in a population of Dutch older adults.

**Methods:**

This cohort study is a secondary analysis of the BCG-CORONA-ELDERLY study, a randomised controlled trial on the effect of BCG vaccination on the cumulative incidence of respiratory tract infections requiring medical intervention in adults ≥60 years. The primary outcome was the cumulative incidence of a self-reported positive SARS-CoV-2 PCR test, and was assessed using a Fine-Gray competing risks model adjusted for baseline characteristics at enrolment. We analysed data from November 1st 2020 until the end of the main study in May 2021.

**Results:**

Routine vaccination data 2020/2021 were available for 1963/2014 (97.5 %) participants; 44/1963 (2.2 %) were excluded due to COVID-19 before vaccination. 1076/1919 (56.1 %) had received the influenza vaccine and 289/1919 (15.1 %) the pneumococcal vaccine. The cumulative incidence of COVID-19 was 0.030 (95 %CI 0.021–0.041) in those vaccinated against influenza compared to 0.029 (95 %CI 0.019–0.041) in the unvaccinated group (subdistribution hazard ratio (SDHR) 1.018; 95 %CI 0.602–1.721). For pneumococcal vaccination the cumulative incidence was 0.031 (95 %CI 0.015–0.056) for the vaccinated and 0.029 (95 %CI 0.022–0.038) for non-vaccinated individuals (SDHR 0.961; 95 %CI 0.443–2.085). BCG vaccination in the previous year and sex were not significant effect modifiers in the primary analysis. Duration of fever, cough and dyspnoea was also not significantly different between treatment arms.

**Conclusion:**

Neither influenza nor pneumococcal vaccination was associated with a lower incidence or shorter duration of COVID-19 symptoms in older adults.

## Background

Recent studies have reported protective heterologous beneficial effects of certain vaccines (e.g. Bacillus Calmette–Guérin (BCG), measles, mumps and rubella vaccine, oral polio vaccine). These vaccines improve the immune response and protection against other infections through heterologous T-cell immunity and non-specific innate immune memory ('trained immunity’) [1].

Older adults experience a decline in adaptive immune responses, and are also at greater risk to have more severe coronavirus disease 2019 (COVID-19), leading to increased hospitalisation and death [2]. For this population-at-risk, vaccines that can induce trained immunity may be useful to decrease disease burden, and could help reduce morbidity and mortality in future outbreaks.

During the SARS-CoV-2 pandemic, there was an increased interest in the heterologous effects of these vaccines for potential protection against severe COVID-19. Large randomised controlled studies using Bacillus Calmette–Guérin (BCG) vaccination were conducted in older adults [3], [4]. Epidemiological studies also reported heterologous protective effects of adjuvanted herpes zoster vaccine against COVID-19 [5].

Every year, at-risk populations such as older adults, are vaccinated against influenza. Several epidemiological studies suggested that the incidence and severity of COVID-19 in individuals who had received the quadrivalent inactivated influenza vaccine (QIV) the year before were lower than in unvaccinated individuals [6], [7]. In vitro, peripheral blood mononuclear cells trained with QIV and restimulated with SARS-CoV-2 produce larger amounts of IL-1RA, an anti-inflammatory cytokine, which in recombinant form is also administered to treat hyperinflammation in COVID-19 [6]. Similarly, one cohort study showed a 52 % reduction in symptomatic COVID-19 after pneumococcal vaccination [7], and another observational study among older adults found an odds ratio of 0.56 (95 % CI 0.33–0.95) for COVID-19 after having received pneumococcal vaccination [8].

Based on this accumulating information, we hypothesised that routine pneumococcal and influenza vaccination might reduce the incidence of COVID-19 in a population of older adults. In this study we assessed the impact of pneumococcal and influenza vaccination on the incidence and duration of symptoms during COVID-19.

## Methods

### Study design

This cohort study is a secondary analysis of the BCG-CORONA-ELDERLY study, a multicentre study in which immunocompetent adults aged 60 years and older were randomised 1:1 to BCG vaccination or placebo to study the effect on the cumulative incidence of respiratory tract infections (RTI) requiring medical intervention [9]. The study was approved by the Arnhem-Nijmegen Ethical Committee (NL73430.091.20). All participants provided written informed consent. The study ran from April 2020 to May 2021 and the follow-up was 12 months in which participants reported health complaints and SARS-CoV-2 PCR test results amongst others. The primary analysis showed no significant differences in the cumulative incidence of clinically relevant RTIs and COVID-19 between those vaccinated with BCG and placebo [3].

Influenza and pneumococcal vaccination took place in the regular immunisation programme during follow-up. The influenza vaccines used in the Netherlands during the study period were all egg-based inactivated split virion vaccines, containing influenza strains A/Victoria/2570/2019, A/Cambodia/e0826360/2020, B/Washington/02/2019, and B/Phuket/3073/2013. The pneumococcal vaccine was a 23-valent polysaccharide vaccine. Influenza vaccination was widely available at no cost for all people over 60 years of age, concomitant pneumococcal vaccination was only offered to those aged 73–79 years, also free of charge. Vaccination campaigns started after participants had been in the trial for approximately 6 months.

### Study participants

Of the initial 2014 participants enrolled in April/May 2020, all 1963 individuals still actively participating in the BCG-CORONA-ELDERLY study provided us with information whether or not they had received the seasonal vaccinations. The information was collected through an electronic questionnaire that was sent in February 2021 (9 months after randomisation). Participants reported information on COVID-19, symptoms (sickness, fever, cough, sore throat, rhinorrhoea, dyspnoea, loss of smell and taste, myalgia, shivering, fatigue, headache and diarrhoea), doctor’s visits, hospitalisation and other infections in a weekly electronic questionnaire. Those who reported COVID-19, infection-related doctor’s visits, or any hospitalisation were contacted by the researchers for confirmation and further information.

### Statistical analysis

We performed a time-to-event analysis using Fine-Gray competing risks analysis. The primary outcome was COVID-19, defined as a self-reported positive SARS-CoV-2 PCR test. To study the possible interaction between BCG vaccination in the previous year and sex with influenza or pneumococcal vaccination, we performed a stratified analysis for these groups. The secondary outcome was duration of symptoms during a COVID-19 episode. The analysis was stratified per vaccine. Individuals who received both vaccines were included in both vaccine cohorts. Adjustment was performed for clinically relevant trial baseline covariates that statistically differed between the two groups based on univariate testing at a significance level of p < 0.05.

COVID-19 symptoms were defined as symptoms 5 days before until 14 days after date of PCR-confirmed SARS-CoV-2 infection and the number of days with symptoms in this period was compared using a Mann-Whitney test.

We also performed an analysis on all intervention groups separately, dividing the cohort in the groups ‘no vaccine’, ‘only influenza vaccine’, ‘only pneumococcal vaccine’, and ‘both vaccines’. The same covariates as in the primary analysis were included (age, sex, cardiovascular disease, and use of metabolic medication).

The exact vaccination date was not known for all participants. Therefore, we only analysed follow-up time November 1st 2020. We chose this date because general practitioners administer both influenza and pneumococcal vaccines mostly in October, as was also confirmed by the known vaccination dates in our study. Follow-up was censored in case of loss to follow-up or at the end of follow-up 12 months after randomisation to BCG or placebo (April–May 2021).

Data was analysed using R version 4.1.1. Statistical significance was defined as p < 0.05.

## Results

### Study participants

Routine vaccination data was available for 1963/2014 (97.5 %) participants. Participants were excluded if they had documented COVID-19 prior to influenza/pneumococcal vaccination on November 1st 2020 (n = 44). Of the remaining 1919 individuals included in the analysis 923 received one of the vaccines only (influenza [n = 855]; pneumococcal [n = 68]) and 221 received both vaccines.

Participants vaccinated against influenza or pneumococcal disease were significantly older, more often had cardiovascular disease and hypertension, used more medication, were more likely to have received an influenza vaccine in the year prior, and more were retired. Additionally, more recipients of a pneumococcal vaccine, but not an influenza vaccine, were using ‘metabolic’ medication (statins, metformin and bisphosphonates), were of the male sex, and had a higher number of daily used medications. The receipt of BCG vaccination was similar between groups (Table 1).

**Table 1:** Baseline characteristics of participants.

Variable	No influenza vaccine (n = 843)	Influenza vaccine (n = 1076)	p-value	No pneumococcal vaccine (n = 1630)	Pneumococcal vaccine (n = 289)	p-value
***Demographic characteristics***						
Mean age (SD) – yr	67.5 (5.5)	68.6 (6.1)	**<0.001**	67.2 (5.6)	73.6 (3.5)	**<0.001**
*Age category – no. ( %)*			**<0.001**			**<0.001**
60–69	585 (69.4)	647 (60.1)		1210 (74.2)	22 (7.6)	
70–79	220 (26.1)	360 (33.5)		320 (19.6)	260 (90.0)	
80+	38 (4.5)	69 (6.4)		100 (6.1)	7 (2.4)	
*Sex*			0.305			**0.004**
Male sex – no. ( %)	454 (53.8)	553 (51.4)		832 (51.0)	175 (60.6)	
Female sex – no. ( %)	389 (46.1)	523 (48.6)		798 (48.9)	114 (39.4)	
Mean BMI (SD) – kg/m^2^	25.8 (4.2)	25.5 (3.7)	0.118	25.7 (4.0)	25.4 (3.4)	0.261
***Medical history***						
*Comorbidities – no. ( %)*						
Cardiovascular disease	133 (15.8)	212 (19.7)	**0.031**	265 (16.3)	80 (27.7)	**<0.001**
Hypertension	236 (28.0)	351 (32.7)	**0.033**	464 (28.5)	123 (42.6)	**<0.001**
Diabetes	54 (6.4)	73 (6.8)	0.811	107 (6.6)	20 (6.9)	0.924
Asthma	49 (5.8)	65 (6.0)	0.910	100 (6.1)	14 (4.8)	0.471
Other pulmonary disease	23 (2.7)	39 (3.6)	0.331	49 (3.0)	13 (4.5)	0.254
*Medication use*						
Use of any medication – no. ( %)	527 (62.5)	800 (74.4)	**<0.001**	1089 (66.9)	238 (82.4)	**<0.001**
Mean number of daily used medication (SD)	3.2 (2.4)	3.3 (2.5)	0.415	3.1 (2.4)	3.8 (2.8)	**<0.001**
Use of ‘metabolic’ medication# – no. ( %)	214 (25.4)	302 (28.1)	0.207	403 (24.7)	113 (39.1)	**<0.001**
*Smoking history – no. ( %)*			0.879			0.168
Never smoked	288 (34.2)	385 (35.8)		567 (34.8)	106 (36.7)	
Past smoking	508 (60.3)	641 (59.6)		973 (59.7)	176 (60.9)	
Current smoking	43 (5.1)	48 (4.5)		84 (5.2)	7 (2.4)	
Passive smoking	3 (0.4)	3 (0.3)		5 (0.3)	0 (0.0)	
Influenza vaccine 2019/20 – no.( %)	391 (46.5)	855 (79.6)	**<0.001**	1024 (62.9)	222 (77.1)	**<0.001**
BCG vaccination in study – no. ( %)	416 (49.3)	535 (49.7)	0.907	800 (49.1)	151 (52.2)	0.353
***Social characteristics***						
*Employment status – no. ( %)*			**0.005**			**<0.001**
Employed	311 (36.9)	319 (29.6)		605 (37.1)	25 (8.7)	
Retired	499 (59.2)	711 (66.1)		948 (58.2)	262 (90.7)	
Unemployed	12 (1.4)	11 (1.0)		22 (1.3)	1 (0.3)	
Other reason not working	21 (2.5)	35 (3.3)		55 (3.4)	1 (0.3)	

# ‘Metabolic’ medication = statins, metformin, or bisphosphonates.

### COVID-19 and related symptoms

We found no significant differences in the cumulative incidence and hazard ratio of a SARS-CoV-2 infection after influenza vaccination (SDHR 1.018 (95 % CI 0.602;1.721)) (Table 2). Neither female sex (interaction hazard ratio (HR) 0.540 (95 % CI 0.184–1.588)) nor BCG vaccination (interaction HR 0.783 (95 % CI 0.271–2.264)) were significant effect modifiers (Table 3, Table 4 respectively). An effect of vaccinations was also not found in the four-group analysis in which individuals who received both vaccinations were analysed separately (see Table S1). The duration of symptoms also did not differ between those who had or had not been vaccinated against influenza (Table 2).

**Table 2:** The effect of routine vaccination on COVID-19 incidence and symptom duration.

Influenza vaccination				
***COVID-19***				
	No influenza vaccination (n = 843)	Influenza vaccination (n = 1076)		
COVID-19 patients - n	24	32		
Cumulative incidence	0.029 (95 % CI 0.019–0.041)	0.030 (95 % CI 0.021;0.041)		
SDHR	[ref]	1.018 (95 % CI 0.602;1.721)		
***Duration of COVID-19 symptoms***				
	No influenza vaccination	Influenza vaccination	Difference(95 % CI)	p-value
Fever – mean days	1.0	1.9	0.9 (-0.3;2.1)	0.199
Cough – mean days	4.6	5.7	1.0 (-2.1;3.8)	0.537
Dyspnoea – mean days	1.0	2.0	1.0 (-0.9;2.9)	0.466
**Pneumococcal vaccination**				
***COVID-19***				
	No pneumococcal vaccination (n = 1630)	Pneumococcal vaccination (n = 289)		
COVID-19 events - n	47	9		
Cumulative incidence	0.029 (95 % CI 0.022;0.038)	0.031 (95 % CI 0.015;0.056)		
SDHR	[ref]	0.961 (95 % CI 0.443;2.085)		
***Duration of COVID-19 symptoms***				
	No pneumococcal vaccination	Pneumococcal vaccination	Difference (95 % CI)	p-value
Fever – mean days	1.5	1.6	0.0 (-1.4;1.5)	0.773
Cough – mean days	5.4	4.3	−1.2 (-3.7;1.4)	0.882
Dyspnoea – mean days	1.7	0.7	−1.3 (-2.5;0.4)	0.780

SDHR – subdistribution hazard ratio.

**Table 3:** The interaction effect of sex on the impact of routine vaccinations on COVID-19 incidence.

Influenza vaccination		
	No influenza vaccination	Influenza vaccination
**Male sex**	n = 454	n = 553
COVID-19 events – n	9	16
Cumulative incidence	0.020 (95 % CI 0.010–0.036)	0.029 (95 % CI 0.017–0.045)
SDHR	[ref]	1.463 (95 % CI 0.647–3.309)
**Female sex**	n = 389	n = 523
COVID-19 events – n	15	16
Cumulative incidence	0.039 (95 % CI 0.023–0.061)	0.031 (95 % CI 0.018–0.048)
SDHR	[ref]	0.791 (95 % CI 0.391–1.598)
**Interaction HR (F/M)**	–	0.540 (95 % CI 0.184–1.588)

**Pneumococcal vaccination**		

	No pneumococcal vaccination	Pneumococcal vaccination

**Male sex**	n = 832	n = 175
COVID-19 events – n	19	6
Cumulative incidence	0.023 (95 % CI 0.023–0.035)	0.034 (95 % CI 0.014–0.069)
SDHR	[ref]	1.512 (95 % CI 0.604–3.785)
**Female sex**	n = 798	n = 114
COVID-19 events – n	28	3
Cumulative incidence	0.035 (95 % CI 0.024–0.050)	0.026 (95 % CI 0.007–0.069)
SDHR	[ref]	0.745 (95 % CI 0.227–2.445)
**Interaction HR (F/M)**	–	0.493 (95 % CI 0.110–2.214)

SDHR – subdistribution hazard ratio HR-hazard ratio F/M – Female/Male.

**Table 4:** The interaction effect of BCG vaccination on the impact of routine vaccinations on COVID-19 incidence.

Influenza vaccination		
	No influenza vaccination	Influenza vaccination
**Placebo**	n = 416	n = 535
COVID-19 events – n	12	18
Cumulative incidence	0.029 (95 % CI 0.016–0.048)	0.034 (95 % CI 0.021–0.052)
SDHR	[ref]	1.080 (95 % CI 0.523–2.231)
**BCG**	n = 427	n = 541
COVID-19 events – n	12	14
Cumulative incidence	0.028 (95 % CI 0.015–0.047)	0.026 (95 % CI 0.015–0.042)
SDHR	[ref]	0.911 (95 % CI 0.422–1.967)
**Interaction HR (B/P)**	–	0.783 (95 % CI 0.271–2.264)

**Pneumococcal vaccination**		

	No pneumococcal vaccination	Pneumococcal vaccination

**Placebo**	n = 800	n = 151
COVID-19 events – n	25	5
Cumulative incidence	0.031 (95 % CI 0.021–0.045)	0.033 (95 % CI 0.021–0.045)
SDHR	[ref]	1.063 (95 % CI 0.406–2.778)
**BCG**	n = 830	n = 138
COVID-19 events – n	22	4
Cumulative incidence	0.027 (95 % CI 0.017–0.039)	0.029 (95 % CI 0.010–0.068)
SDHR	[ref]	1.092 (95 % CI 0.377–3.159)
**Interaction HR (B/P)**	–	1.026 (95 % CI 0.244–4.303)

SDHR – subdistribution hazard ratio HR – hazard ratio B/P – BCG/Placebo.

For pneumococcal vaccination the cumulative incidences were also similar in both groups (SDHR 0.961 (95 % CI 0.443;2.085)) and symptom duration did not significantly differ (Table 2). Neither female sex (interaction HR 0.493 (95 % CI 0.110–2.214)) nor BCG (interaction HR 1.026 (95 % CI 0.244–4.303)) significantly modified vaccine effects (Table 3, Table 4 respectively).

Severe COVID-19 leading to hospitalisation or death was rare in this cohort. Only 2 people were hospitalised for COVID-19 during the follow-up considered in this post-hoc analysis. One of these individuals received a placebo vaccination at trial enrolment, and flu vaccination, but not a pneumococcal vaccine. This participant was admitted into the ICU with respiratory support for COVID-19. The other participant received a BCG vaccine at trial enrolment, but neither flu nor pneumococcal vaccine and was only shortly hospitalised. We found no effect of influenza and pneumococcal vaccination on hospitalisation for any reason, nor on (self-reported) infections other than COVID-19 (Supplementary Table 2 and 3 respectively).

## Discussion

In our observational post-hoc analysis of a randomized BCG intervention trial in a population of Dutch older adults neither quadrivalent inactivated influenza nor 23-valent pneumococcal vaccination offered heterologous protection against COVID-19 in the approximately 6 months after routine vaccination during the 2020/2021 winter and spring. These vaccinations were also not associated with shorter COVID-19 symptom duration. Neither sex nor BCG were significant effect modifiers, although confidence intervals were too broad to fully exclude relevant effect modification.

All observational studies are prone to bias in the relationship between determinant and outcome. However, the prospective design of our study, which included all individuals at risk for COVID-19 and not just those who had been tested for COVID-19, avoided selection biases that occurred in other vaccine studies [10]. Systematic bias due to lack of accurate measurements (information bias) was also unlikely in our study. Indeed, we asked subjects for their information shortly after vaccinations, making it unlikely that selective reporting would have occurred. Moreover, presumably everyone was equally inclined to be tested for COVID-19, since this was a nested sub-study in which everyone was equally encouraged to get tested for (mild) symptoms because of the weekly mandatory questions on symptoms and COVID-19 testing.

Participants who received the influenza and pneumococcal vaccination had more comorbidities and were older, which is the result of the lack of randomisation for this sub-study. Consequently, they may have felt more vulnerable, which may have made them more compliant with social distancing recommendations. Furthermore, they had fewer work-related contacts. This may have led to association bias due to confounding, however, we adjusted for any statistically significant differences in the analysis. Another limitation is that the study was not designed to assess the effects of influenza and pneumococcal vaccination and is therefore underpowered. There were only limited cases of COVID-19, but no trend was even apparent in any direction.

Our data contrast with other studies that reported a significant reduction in SARS-CoV-2 incidence and severity after influenza vaccination [6], [7]. There are various potential reasons why our study shows a different result. As discussed above, this study is distinguished from the others by its prospective design in which the symptoms of COVID-19 were recorded during the disease episode itself. In addition, differences in the included population may explain the observed differences. Previous studies focused on adults of working age [11], [12] and children [7], whereas we included older people. Declining immune function in the elderly could explain the lack of vaccine effectiveness [13]. Other biological explanations could lie in the type of vaccine and viral infection studied. In previous murine and human studies BCG vaccination seemed to protect against influenza, but not COVID-19 [3], [14]. This may well be true for influenza and pneumococcal vaccinations too.

Even though we did not find significant differences, it is important not to reject the potential impact of routine vaccination on health. During the COVID-19 pandemic many vaccination programmes were halted or delayed while health care systems learned to cope with the pandemic, leading to additional burdens of disease and threats of outbreak of infectious diseases [15]. Future studies should shed light on which existing vaccines have the potential to boost the immune response against heterologous (and emerging) pathogens.

## Declaration of Competing Interest

The authors declare the following financial interests/personal relationships which may be considered as potential competing interests: Cornelis van Werkhoven received support from DaVolterra, bioMérieux, and LimmaTech (payments made to UMC Utrecht), and consulting fees from Merck/MSD and Sanofi-Pasteur (payments made to UMC Utrecht). Mihai Netea was supported by an ERC Advanced Grant (833247) and Fast Grant COVID19. Reinout van Crevel received a grant from the National Institute of Health (R01AI165271), and participated in the Data Safety Monitoring Board of unrelated tuberculosis trials.

## Data Availability

Data can be made available upon reasonable request.
