# Supplementary material for: Routine vaccination for influenza and pneumococcal disease and its effect on COVID-19 in a population of Dutch older adults
Source: Vaccine X. 2023 Jul 6;14:100344. doi: 10.1016/j.jvacx.2023.100344 (PMC10362126; doi:10.1016/j.jvacx.2023.100344)
Supplement: Supplementary Tables [file mmc1.docx]

**Supplemental information**

**Table S1: Results of the Fine-Gray competing risks model assessing the association between the receipt of one, both or no vaccination and self-reported positive SARS-CoV-2 PCR test.**

| **COVID-19** | | | | |
| --- | --- | --- | --- | --- |
|  | No vaccine  (n=775) | Only  influenza vaccine  (n=855) | Only pneumococcal vaccine  (n=68) | Both vaccines  (n=221) |
| COVID-19 events - n | 23 | 24 | 1 | 8 |
| Cumulative incidence | 0.030  (95% CI 0.019-0.043) | 0.028  (95% CI 0.019-0.041) | 0.015  (95% CI 0.001-0.071) | 0.036  (95% CI 0.017-0.067) |
| SDHR | [ref] | 0.919  (95% CI 0.517-1.631) | 0.428  (95% CI 0.057-3.236) | 1.074  (95% CI 0.460-2.511) |

SDHR – subdistribution hazard ratio

**Table S2: The effects of routine vaccination on all-cause hospitalisation**

| **Influenza vaccination** | | |
| --- | --- | --- |
|  | No influenza vaccination  (n=843) | Influenza vaccination  (n=1076) |
| ***Hospitalisation for any reason*** | | |
| Events – n | 9 | 20 |
| Cumulative incidence | 0.011 (95% CI 0.005-0.020) | 0.019 (95% CI 0.012-0.028) |
| SDHR | [ref] | 1.599 (95% CI 0.717-3.569) |
| **Pneumococcal vaccination** | | |
|  | No pneumococcal vaccination  (n=1630) | Pneumococcal vaccination  (n=289) |
| ***Hospitalisation for any reason*** | | |
| Events – n | 20 | 9 |
| Cumulative incidence | 0.012 (95% CI 0.008-0.019) | 0.031 (95% CI 0.015-0.056) |
| SDHR | [ref] | 1.837 (95% CI 0.799-4.223) |

SDHR – subdistribution hazard ratio

| **Influenza vaccination** | | |
| --- | --- | --- |
|  | No influenza vaccination  (n=843) | Influenza vaccination  (n=1076) |
| ***Respiratory tract infection*** | | |
| Events - n | 21 | 35 |
| Cumulative incidence | 0.025 (95% CI 0.016-0.037) | 0.033 (95% CI 0.023-0.044) |
| SDHR | [ref] | 1.291 (95% CI 0.741-2.251) |
| ***Skin infection*** | | |
| Events - n | 18 | 32 |
| Cumulative incidence | 0.021 (95% CI 0.013-0.033) | 0.030 (95% CI 0.021-0.041) |
| SDHR | [ref] | 1.407 (95% CI 0.788-2.511) |
| ***Urinary tract infection*** | | |
| Events - n | 15 | 28 |
| Cumulative incidence | 0.018 (95% CI 0.010-0.029) | 0.026 (95% CI 0.018-0.037) |
| SDHR | [ref] | 1.339 (95% CI 0.734-2.669) |
| ***Gastro-intestinal infection*** | | |
| Events - n | 6 | 10 |
| Cumulative incidence | 0.007 (95% CI 0.003-0.015) | 0.009 (95% CI 0.005-0.017) |
| SDHR | [ref] | 1.276 (95% CI 0.451-3.611) |
| **Pneumococcal vaccination** | | |
|  | No pneumococcal vaccination  (n=1630) | Pneumococcal vaccination  (n=289) |
| ***Respiratory tract infection*** | | |
| Events - n | 46 | 10 |
| Cumulative incidence | 0.029 (95% CI 0.021-0.038) | 0.035 (95% CI 0.018-0.061) |
| SDHR | [ref] | 1.072 (95% CI 0.478-2.401) |
| ***Skin infection*** | | |
| Events - n | 43 | 7 |
| Cumulative incidence | 0.028 (95% CI 0.020-0.038) | 0.024 (95% CI 0.011-0.047) |
| SDHR | [ref] | 0.817 (95% CI 0.342-1.949) |
| ***Urinary tract infection*** | | |
| Events - n | 36 | 7 |
| Cumulative incidence | 0.022 (95% CI 0.016-0.030) | 0.024 (95% CI 0.011-0.047) |
| SDHR | [ref] | 0.875 (95% CI 0.365-2.099) |
| ***Gastro-intestinal infection*** | | |
| Events - n | 14 | 2 |
| Cumulative incidence | 0.009 (95% CI 0.005-0.014) | 0.007 (95% CI 0.001-0.023) |
| SDHR | [ref] | 0.921 (95% CI 0.204-4.169) |

**Table S3: The effect of routine vaccination on self-reported infections diagnosed by a physician**

SDHR – subdistribution hazard ratio
